# Narrow-Complex Tachycardia With Intermittent Irregularity and Variable QRS Morphology

**DOI:** 10.1016/j.jaccas.2026.107727

**Published:** 2026-05-20

**Authors:** Juan Mundisugih, Rajiv Mahajan

**Affiliations:** a Department of Cardiology, Westmead Hospital, Westmead, New South Wales, Australia; b Sydney Medical School, The University of Sydney, Sydney, New South Wales, Australia; c Department of Cardiology, Lyell McEwin Hospital, Elizabeth Vale, South Australia, Australia; d School of Medicine, College of Health, Adelaide University, Adelaide, South Australia, Australia

**Keywords:** atrial fibrillation, narrow-complex, ventricular tachycardia

## Abstract

**Case Summary:**

We describe a case of an 83-year-old patient presenting with palpitations and a serial electrocardiogram demonstrating narrow-complex tachycardia with varying regularity and QRS morphology. This case highlights the diagnostic value of serial electrocardiogram analysis and detailed QRS morphological assessment in elucidating underlying arrhythmia mechanisms, enabling accurate diagnosis of complex tachyarrhythmias.

**Take-Home Messages:**

Serial electrocardiogram analysis, particularly tracking QRS morphology changes, provides critical insights beyond pattern recognition in arrhythmia diagnosis. Although common conditions present familiar patterns, detailed morphological evaluation reveals underlying mechanisms, enabling precise identification of complex arrhythmias and guiding targeted interventions.

## Visual Summary

**Figure:**
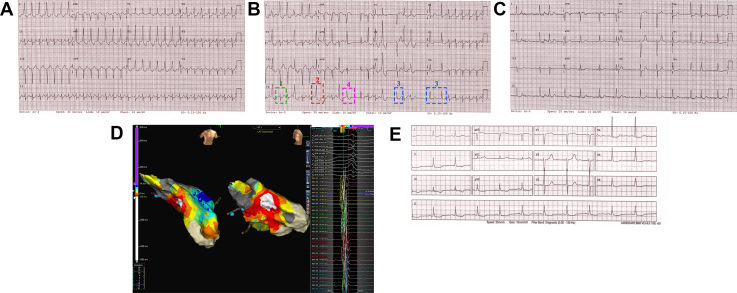

## Case Summary

**Figure 1:**
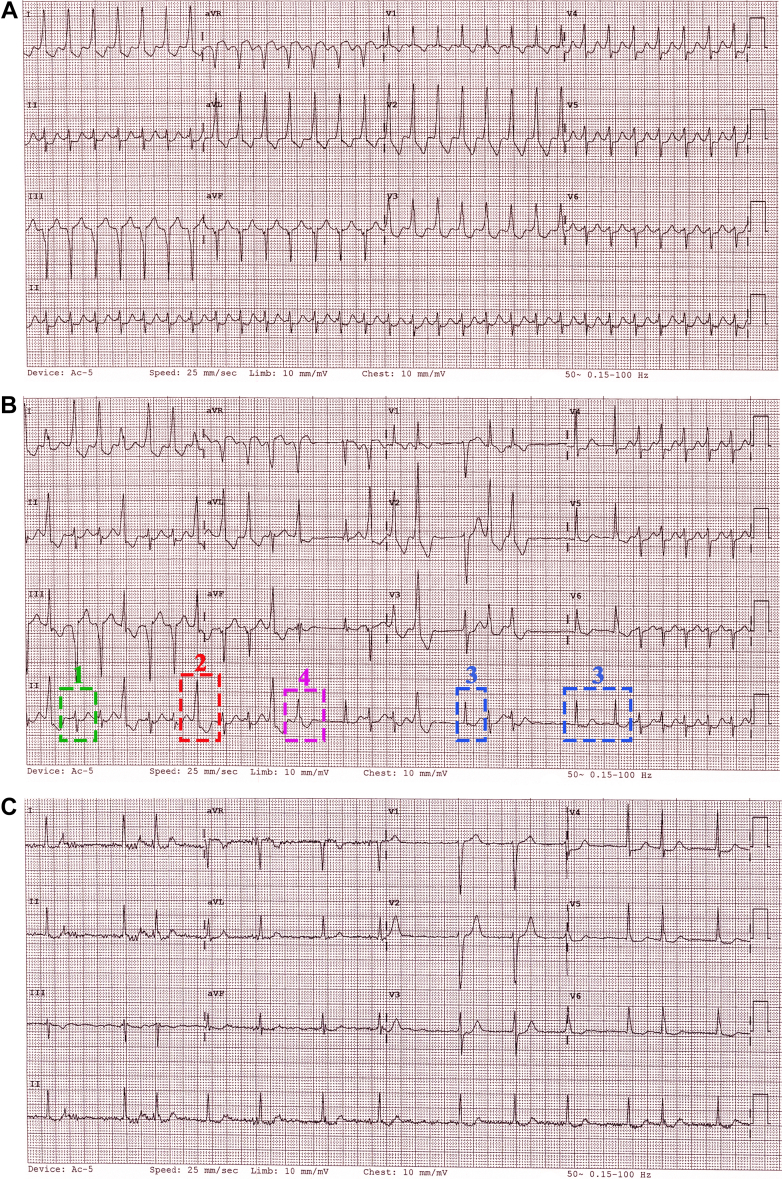

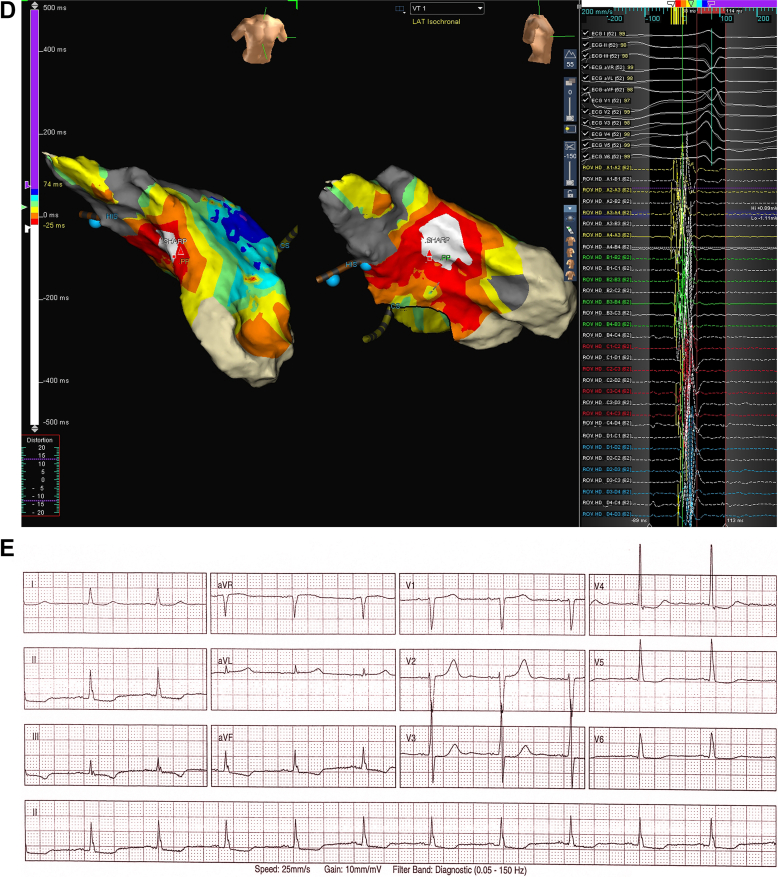
Serial Electrocardiograms and Electroanatomical Mapping in a Patient With Narrow-Complex Tachycardia Exhibiting Intermittent Irregularity and Variable QRS Morphology Serial ECGs demonstrate evolution from (A) regular tachycardia with left axis deviation and right bundle branch block-like morphology, to (B) intermittent irregularity with multiple QRS morphologies, and progression to (C) irregular rhythm with incomplete left bundle branch block pattern. (D) Electroanatomical mapping localized earliest activation to the basal mid-septal region consistent with the left posterior fascicle. (E) Postablation ECG shows sinus rhythm with incomplete left bundle branch block pattern. ECG = electrocardiogram.

An 83-year-old man presented with persistent palpitations. His history included gastroesophageal reflux, hypertension, and dyslipidemia. Vital signs and physical examination findings were normal. Investigations showed negative troponin levels and normal routine blood test results. A transthoracic echocardiogram showed normal left ventricular function, no regional wall motion abnormalities, mildly enlarged left atrium, and normal valvular function. Serial electrocardiograms (ECGs) were obtained (Figures 1A to 1C) within 30 minutes.

**Take-Home Messages**

• Serial electrocardiogram analysis, particularly tracking QRS morphology changes, provides critical insights beyond pattern recognition in arrhythmia diagnosis.
• Although common conditions present familiar patterns, detailed morphological evaluation reveals underlying mechanisms, enabling precise identification of complex arrhythmias and guiding targeted interventions.

What is the most likely diagnosis?

1. Atrial fibrillation (AF)/flutter with intermittent aberrancy/pre-excitation
2. Atrioventricular nodal re-entrant tachycardia (AVNRT) with underlying AF
3. Atrial tachycardia progressing to AF
4. Concomitant ventricular tachycardia (VT) and AF.

## Discussion

Serial ECGs show evolving arrhythmias. Figure 1A shows regular tachycardia (176 beats/min) with left axis deviation and right bundle branch block–like morphology. Figure 1B shows intermittent irregularity with 4 QRS morphologies: 1) the original pattern; 2) every-third-beat wide complexes; 3) narrow complexes with incomplete left bundle branch block; and 4) fusion beats. Figure 1C shows progression to an irregular rhythm with an incomplete left bundle branch block pattern, consistent with AF.

Viewed alone, the alternating narrow-wide tachycardia in Figure 1B may suggest AF with pre-excitation. However, the absence of delta waves in Figure 1C makes AF with pre-excitation unlikely. The transition from a regular to an irregular rhythm, with suspicious flutter waves buried within the QRS complexes in Figure 1A, may suggest atrial tachycardia/flutter degenerating into AF. However, this diagnosis becomes less probable given the intermittent group beating, intermittent regularity, and the distinct QRS variations in Figure 1B. In addition, the wide QRS complex (QRS #2) in Figure 1B is consistent with premature ventricular complexes, showing an inferior axis and limb lead pattern (I-positive, aVL/aVR-negative) suggestive of a ventricular outflow tract origin. All these findings suggest a complex mechanism with competing rhythms and underlying AF, rather than a transition between arrhythmias.

Arrhythmias coexisting with AF must originate outside the chaotic atria—typically from the atrioventricular (AV) node, conduction system, or ventricles. Although the regular, relatively narrow tachycardia may represent AVNRT, its spontaneous reinitiation without triggers and very brief duration (2 beats) favor automaticity, despite the known coexistence of AVNRT and AF.^1^ The relatively narrow QRS complex in Figure 1A suggests a conduction system origin. Right bundle branch block–like morphology with a left-superior axis, positive QRS complexes in V_3_-V_4_, late precordial transition, and an rS pattern in V_6_ suggests left posterior fascicular VT.^2^ The absence of hidden P waves and any evidence of AV dissociation is due to the underlying AF.

An electrophysiology study was performed for the recurrent tachycardia. The ECG morphology during tachycardia had alternated between Figures 1A to 1C overnight. During the procedure, the patient was in the tachycardia shown in Figure 1A and hemodynamically stable. Baseline rhythm showed irregular A-A and regular V-V intervals. After cardioversion, 2 sinus beats were seen before tachycardia recurred with AV dissociation and V preceding His signal, confirming VT. Electroanatomical mapping identified earliest activation at the basal midseptal region, corresponding to the left posterior fascicular area (Figure 1D). Pacing in this area produced an identical QRS complex to Figure 1A. Radiofrequency ablation successfully terminated the VT, with postprocedure ECG showing sinus rhythm with a third QRS pattern (Figure 1E).

## Funding Support and Author Disclosures

Dr Mundisugih is supported by an Australian Government Research Training Program Scholarship. The Adelaide University reports receiving consulting fees from Abbott on behalf of Dr Mahajan. The Adelaide University also reports receiving research funding from Abbott and Medtronic on behalf of Dr Mahajan.
